# CT and MRI Manifestations of Pancreatic Hemangioma: A Literature Review and a New Case Report

**DOI:** 10.1002/ccr3.71914

**Published:** 2026-01-22

**Authors:** Jing Zhang, Jingjing Chen, Dongmei Zou, Xu Cao

**Affiliations:** ^1^ Department of Radiology The People's Hospital of Shifang Deyang Sichuan China; ^2^ Department of Pathology The People's Hospital of Shifang Deyang Sichuan China; ^3^ Department of Ultrasound Medicine The People's Hospital of Shifang Deyang Sichuan China

**Keywords:** case report, CT, hemangioma, MRI, pancreas

## Abstract

Pancreatic hemangioma is an extremely rare benign tumor occurring in the pancreas. Preoperative diagnosis is challenging due to its lack of specificity in clinical manifestations and imaging examinations. A pancreatic lesion was found during physical examination of a 54‐year‐old woman who had no symptoms of abdominal discomfort. Computed tomography (CT) revealed a round, well‐defined, and low‐density lesion in the head of the pancreas with a multilocular or honeycomb shape, mild–moderate enhancement of its internal septum, and no enhancement of the cystic component. Magnetic resonance imaging (MRI) showed an abnormal signal lesion in the head of the pancreas, with isointensity and hypointensity on T1‐weighted images and inhomogeneous hyperintensity on T2‐weighted images. The isointensity on diffusion weighted imaging (DWI) and high apparent diffusion coefficient (ADC) values suggested no significant diffusion restriction. In addition, the main pancreatic duct was not dilated and did not communicate with the lesion. Clinical consideration of pancreatic serous cystadenoma was highly probable. However, a pancreatic hemangioma was confirmed postoperatively.

## Summary

The atypical imaging manifestations of pancreatic hemangioma make it difficult to differentiate it from other lesions.

Multimodal imaging is of great value in the diagnosis and treatment decision‐making of pancreatic hemangiomas.

**Abbreviations**
ADC: apparent diffusion coefficient
AFP: alpha‐fetoprotein
CA125: carbohydrate antigen 125
CA199: carbohydrate antigen 199
CEA: carcinoembryonic antigen
CT: computed tomography
DWI: diffusion weighted imaging
ERCP: endoscopic retrograde cholangiopancreatography
EUS: endoscopic US
EUS‐FNA: Endoscopic Ultrasound‐guided Fine Needle Aspiration
IPMN: intraductal papillary mucinous neoplasms
MRI: magnetic resonance imaging
US: ultrasonography

## 1 Introduction

Hemangiomas result from the rapid proliferation of endothelial cells in early infancy and then degenerate over time, which are relatively common benign tumors that can occur anywhere in the body and are most commonly found under the skin of the head, neck, and trunk [1]. Among the internal organs, the liver and spleen are common sites where hemangiomas occur. However, hemangiomas occurring in the pancreas are extremely rare [2]. Pancreatic hemangioma is poorly recognized and difficult to diagnose preoperatively because of its low incidence, varied clinical presentation, and atypical imaging findings. Therefore, most lesions are diagnosed incidentally after surgery.

We recently encountered a woman with an occupancy in the head of the pancreas, which was proposed to be diagnosed as a serous cystadenoma on preoperative computed tomography (CT) and magnetic resonance imaging (MRI) but turned out to be diagnosed as pancreatic hemangioma postoperatively. Therefore, we analyzed the imaging findings of this case of pancreatic hemangioma and reviewed the literature to deepen our understanding of this rare disease.

## 2 Case History/Examination

**FIGURE 1:**
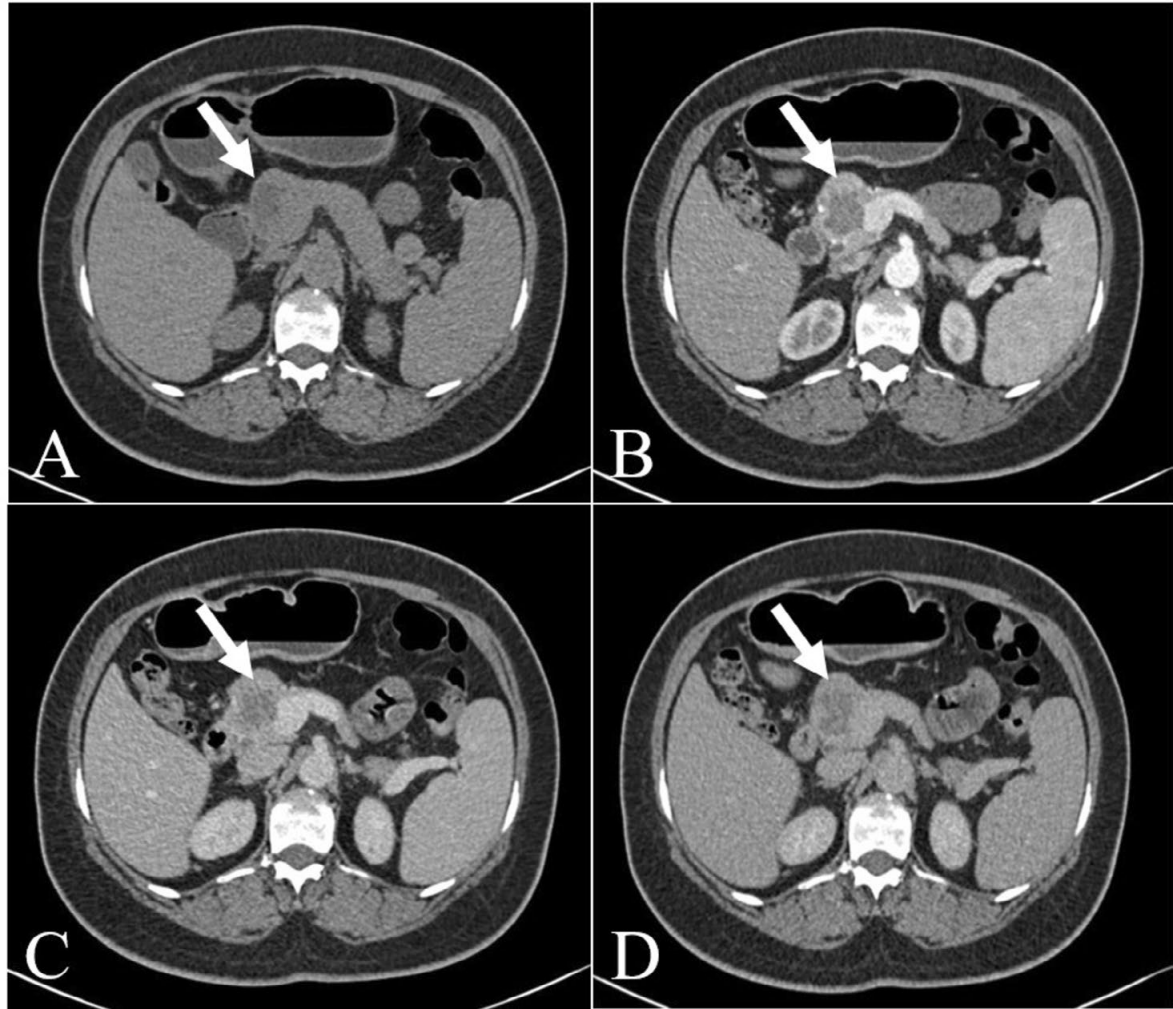
Non‐enhanced computed tomography (CT) revealed a round, well‐defined, and low‐density lesion in the head of the pancreas, measuring about 2.5 × 1.5 × 2.0 cm. Additionally, no significant calcification was observed (A). Enhancement scans showed internal segments of the lesion with mild to moderate enhancement and internal cystic components with no enhancement, which appear as a multilocular or honeycomb shape (B–D).

**FIGURE 2:**
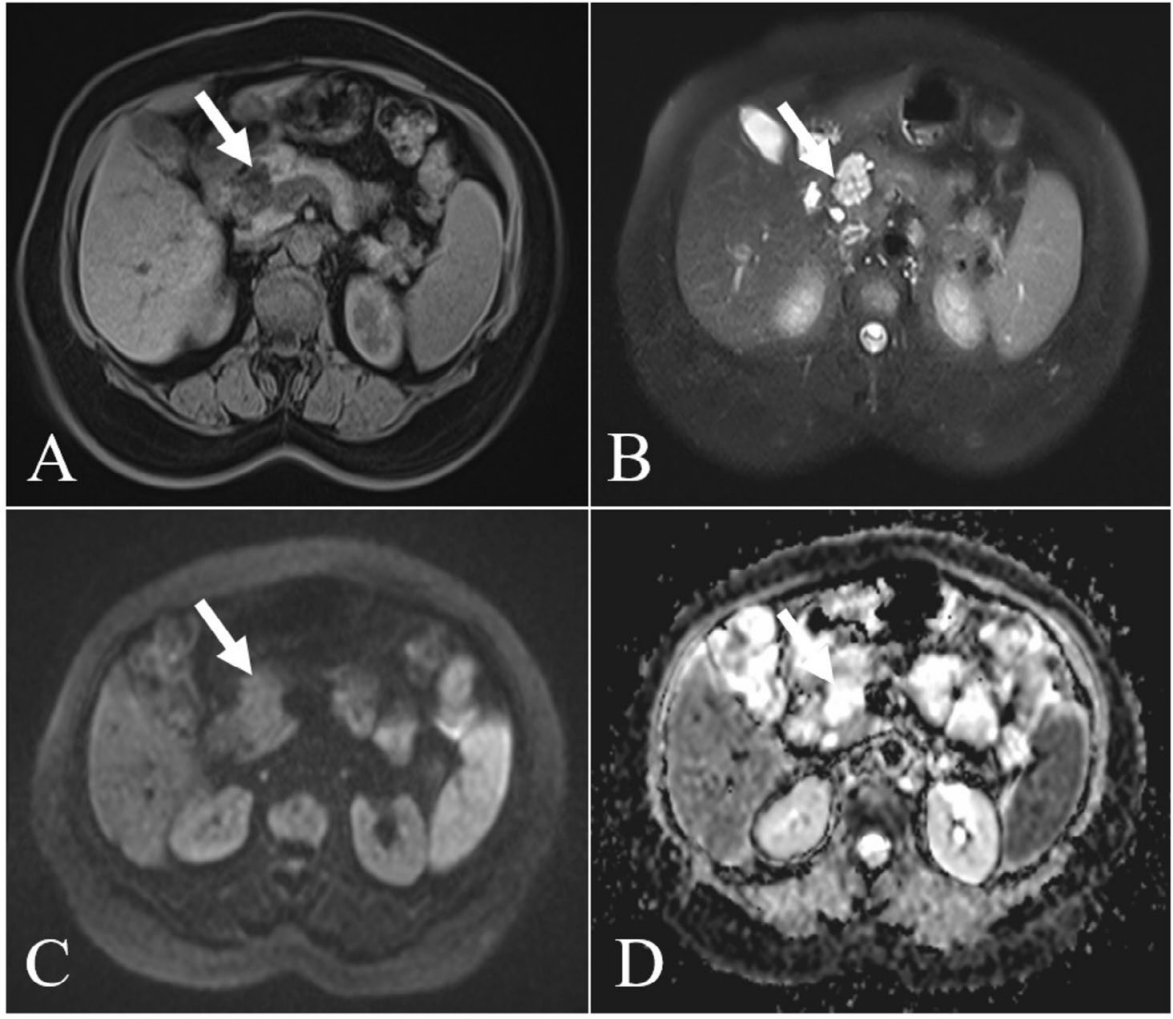
Magnetic resonance imaging (MRI) showed an abnormal signal nodule in the head of the pancreas, with isointensity and hypointensity on T1‐weighted images (A) and inhomogeneous hyperintensity on T2‐weighted images (B). The isointensity on diffusion weighted imaging (DWI; C) and high apparent diffusion coefficient (ADC; D) values indicated a low impedance to diffusion of water molecules within the lesion. In addition, the lesion was not connected to the main pancreatic duct and there was no dilatation of the main pancreatic duct.

A 54‐year‐old female was transferred to our hospital due to a lesion in the head of the pancreas accidentally found by ultrasonography (US) during physical examination in an outside hospital. Subsequently, she was treated with medication (details unknown) and came to our hospital for review. Except for the past history of hepatitis B and hysterectomy, any symptoms of abdominal discomfort were denied by the patient. Laboratory tests after admission showed three positives: hepatitis B surface antigen (+), anti‐HBe (+), and anti‐HBc (+). In addition, tumor markers, including carcinoembryonic antigen (CEA), alpha‐fetoprotein (AFP), carbohydrate antigen 125 (CA125), carbohydrate antigen 199 (CA199), and other routine laboratory tests were within normal ranges. Non‐contrast CT revealed a round, well‐defined, and low‐density lesion in the head of the pancreas, measuring about 2.5 × 1.5 × 2.0 cm. Additionally, no significant calcification was observed. Enhancement scans showed internal segments of the lesion with mild to moderate enhancement and internal cystic components with no enhancement, which appear as a multilocular or honeycomb shape (Figure 1A–D). MRI showed an abnormal signal lesion in the head of the pancreas, with isointensity and hypointensity on T1‐weighted images and inhomogeneous hyperintensity on T2‐weighted images. The isointensity on diffusion weighted imaging (DWI) and high apparent diffusion coefficient (ADC) values indicated low impedance to the diffusion of water molecules within the lesion. In addition, the lesion was not connected to the main pancreatic duct and there was no dilatation of the main pancreatic duct (Figure 2A–D). Finally, a combination of preoperative clinical and imaging tests led to the diagnosis of a serous cystadenoma of the pancreatic head.

## 3 Differential Diagnosis, Investigations and Treatment

Clinically, pancreatic hemangiomas are easily confused with other common cystic pancreatic lesions such as pseudocysts, serous cystadenomas, mucinous cystadenomas, and intraductal papillary mucinous neoplasms (IPMN). Pancreatic pseudocysts do not enhance on contrast‐enhanced CT scans, and patients often have a history of chronic pancreatitis. Serous cystadenomas predominantly occur in elderly women and typically present on imaging as cystic or lobulated lesions with septa, which may calcify. Mucinous cystadenomas are more common in middle‐aged women, appearing as smooth‐edged lesions with or without septa, often featuring peripheral eggshell calcifications. IPMN presents as pleomorphic cystic masses communicating with the main pancreatic duct, which is key to diagnosis. Beyond these, numerous other less common pancreatic tumors present as cystic lesions. These include pancreatic solid pseudopapillary neoplasms, cystic neuroendocrine tumors, cystic degeneration of other solid tumors, and extremely rare cystic acinar cell tumors, ductal tubular neoplasms, hemangiomatous tumors, lymphoepithelial cysts, and a few other mesenchymal‐derived tumors.

Preoperative clinical evaluation strongly suggested the pancreatic serous cystadenoma, though a definitive diagnosis was not established. Additionally, the patient experienced significant psychological distress and expressed a strong desire for surgery. Indeed, patients should be advised to undergo Endoscopic Ultrasound‐guided Fine Needle Aspiration (EUS‐FNA) in accordance with the guidelines. Our clinicians also made this recommendation. However, due to the uncertainty and risk of diagnosis of EUS‐FNA, and the patient's unwillingness to refer to a higher hospital and bear the additional costs of EUS‐FNA, she rejected this suggestion and chose direct surgery. At the patient's request and after the surgical contraindications were ruled out, a laparoscopic pancreaticoduodenectomy was performed.

## 4 Conclusion and Results (Outcome and Follow‐Up)

**FIGURE 3:**
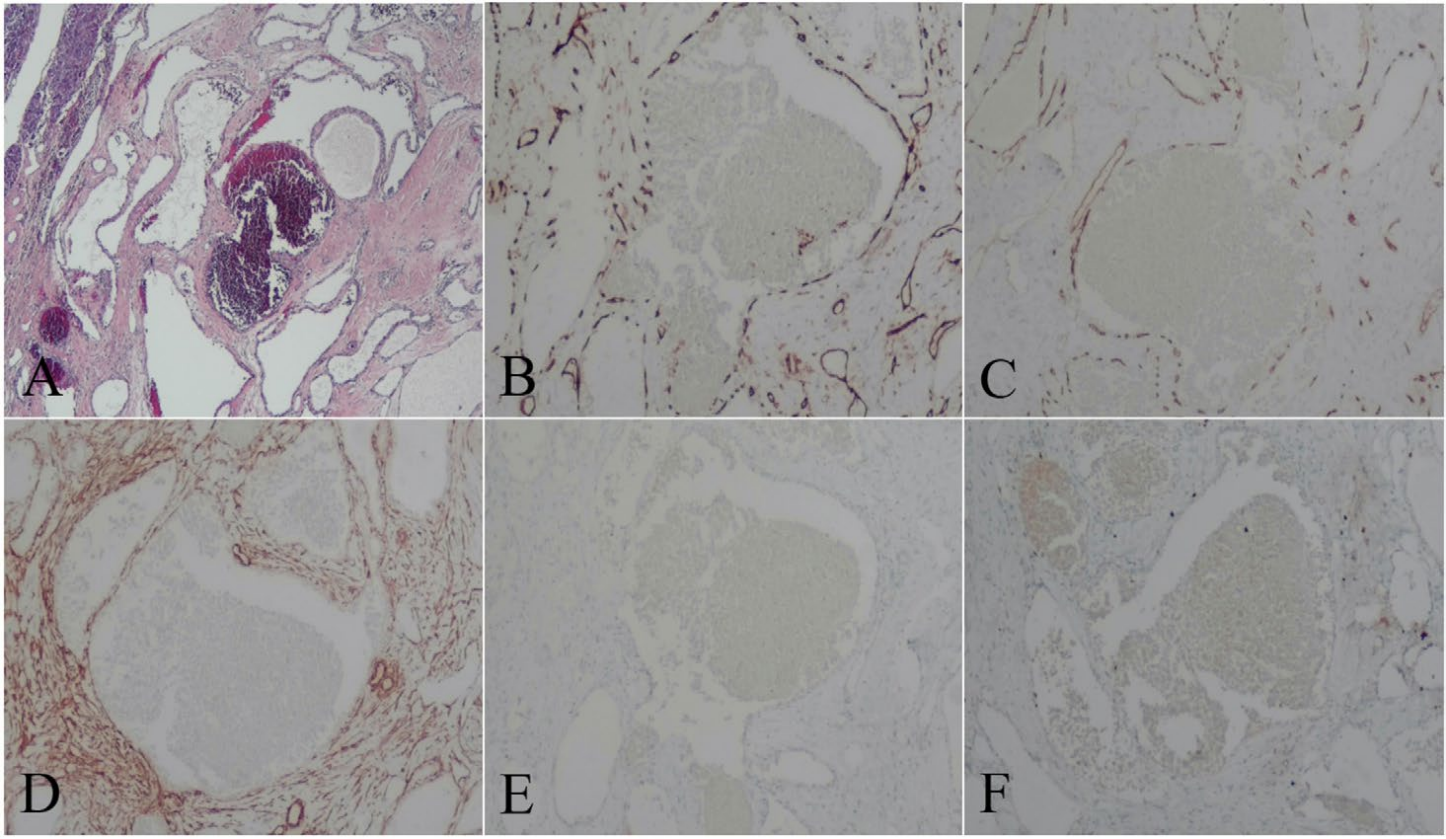
Postoperative light microscopy showed that the mass consisted of dilated blood vessels of different diameters, and it was considered a tumor of vascular origin in the head of the pancreas, mostly cavernous hemangioma (A). Immunohistochemical staining showed: CD31(+; B), CD34(+; C), SMA(+; D), D2‐40(−; E), Ki‐67(−; F), supporting the diagnosis of cavernous hemangioma.

A solid mass in the pancreatic head was observed on the postoperative autopsy specimen, and the mass appeared gray and white on the cut surface. Postoperative light microscopy showed that the mass consisted of dilated blood vessels of different diameters, and it was considered a tumor of vascular origin in the head of the pancreas, most likely a cavernous hemangioma (Figure 3A). Immunohistochemical staining showed: CD31(+), CD34(+), SMA(+), D2‐40(−), Ki‐67(−) (Figure 3B–F). The final postoperative pathological diagnosis was cavernous hemangioma. The patient had no complications during the clinical follow‐up 4 months after surgery. No signs of recurrence or metastasis were found on CT examination of the upper abdomen.

## 5 Discussion

Since 1939, a total of 30 reports of pancreatic hemangioma have been retrieved from PubMed. In addition to our report this time, a total of 34 cases (Table 1) of pancreatic hemangioma were analyzed [3, 4, 5, 6, 7, 8, 9, 10, 11, 12, 13, 14, 15, 16, 17, 18, 19, 20, 21, 22, 23, 24, 25, 26, 27, 28, 29, 30, 31, 32]. According to these cases, like other hemangiomas, pancreatic hemangiomas were also more common in women (26 cases in this series, 76.5%), with a male to female sex ratio of 1.0:3.3. The most common symptom remains abdominal pain (21/34, 61.8%), which usually occurs in the upper abdomen and sometimes radiates to the back. Other associated symptoms include low back pain, bloating, nausea, and vomiting, all caused by the tumor pressing on surrounding organs. Others were incidental clinical findings or physical examination findings.

**TABLE 1:** Literature review of adult pancreatic hemangiomas.

No.	Year	Author	Age/sex	Clinical symptom	Imaging examination	Clinical diagnosis	Site/size (cm)	Treatment	Follow up
1	1939	Ranstrom [3]	61/F	—	Found at autopsy	—	Head/7 × 7	—	—
2	1961	Ringoir et al. [4]	71/F	Melena	Plain abdominal film, transvenous cholangiography	—	Head/15 × 15	Retrocolic gastroenterostomy, vagotomy	—
3	1972	Colardyn et al. [5]	42/F	Upper abdominal pain radiating to the back	Plain abdominal film, angiography	—	Body tail/−	Fat free diet, anticholinergic	—
4	1985	Mangin et al. [6]	62/F	Abdominal discomfort, vomiting	US, ERCP, CT, angiography	—	Head to tail/20 × 7	Laparotomy with observation	—
5	1991	Kobayashi et al. [7]	30/M	Abdominal bloating	US, CT, MRI, angiography	—	Head/maximum diameter 20	Pancreatoduodenectomy	—
6	1991	Dageförde et al. [8]	79/F	Abdominal pain	US, ERCP, angiography	—	Body tail/6 × 3	Observation	—
7	2003	Chang et al. [9]	70/F	Fever, chills, productive cough, and vague epigastralgia	CT, angiography	Serous/cystic adenoma/adenocarcinoma	Body tail/4.0 × 3.2 × 3.0	Subtotal pancreatectomy	No signs of recurrence or metastasis 14 months after surgery
8	2006	Plank et al. [10]	36/M	Abdominal pain	CT, MRI, intraoperative US	—	Head/maximum diameter 3	Not resected	—
9	2008	Xu et al. [11]	60/F	Upper abdominal pain, fever	US, CT	Adenoma/adenocarcinoma	Tail/2 × 2	Pancreas body tail resection + splenectomy	Successful recovery and healthy survival after surgery
	2008	Xu et al.	41/F	No obvious clinical symptoms	US	Adenoma/islet cell carcinoma	Body/2.5 × 2.0	Partial resection of the body of the pancreas	
	2008	Xu et al.	30/F	Upper abdominal pain, obstruction	US	Benign and cystic tumor	Head/6 × 5	Pancreaticoduodenectomy	
10	2009	Mundinger et al. [12]	45/F	Radiating pain in the left upper abdomen	CT, MRI	—	Head/6.2 × 5.3	Frozen section	—
11	2011	Weidenfeld et al. [13]	73/F	Radiating pain in the upper abdomen	CT	—	Head/5.5 × 4.0 × 3.0	Whipple's procedure	—
12	2011	Lee et al. [14]	49/F	Vertigo, palpitation	US, intraoperative US, CT	Mucious cyst with malignant features	Neck/5	Central partial pancreatectomy and gastrostomy	Symptoms disappeared after 6 months
13	2012	Kersting et al. [15]	53/M	No symptoms	US, CT, MRI	Adenocarcinoma	Head/8	Extirpation of the tumor	—
14	2013	Malik et al. [16]	70/F	—	CT	—	Head/8	Pylorus preserving pancreatoduodenectomy	—
15	2013	Lu and Wu [17]	23/F	No symptoms	US, CT, MRI	—	Head/5.0 × 4.0 × 3.1	—	—
16	2014	Williamson et al. [18]	78/F	Increasing epigastric pain	EUS, CT	—	Head/4	Observation	Repeat CT at four and ten months was unchanged
17	2014	Naito et al. [19]	40/F	Abdominal pain	CT, MRI	Multilocular septated cystic mass	Body tail/10	Pancreatectomy	No recurrence 6 years after surgery
18	2014	Kim et al. [20]	48/F	Incidentally detected	CT, MRI	Neuroendocrine tumor	Tail/0.5 × 0.6	Distal pancreatectomy	—
	2014	Kim et al.	53/F	Abdominal pain	CT, MRI	Cystic mass	Body/−	Distal pancreatectomy	—
19	2015	Mondal et al. [21]	18/F	Epigastric pain	US, CT, MRI	Benign cyst	Head/5 × 6	Pylorus preserving pancreatoduodenectomy	No symptoms 6 months after surgery
20	2015	Lu and Yang [22]	28/F	Abdominal pain	CT	Serous cystadenoma or pseudocyst	Body tail/5.5 × 6.5 × 5.5	Subtotal pancreatectomy and splenectomy	Without any signs of recurrence; follow‐up was for 2 years and 4 months.
21	2015	Soreide et al. [23]	38/F	Left epigastric pain, nausea, palpable left subcostal mass	US, MRI	Solid pseudopapillary epithelial neoplasm	Tail/19.5 × 10 × 7	Distal pancreatectomy, splenectomy	No complaints or concerns
22	2016	Bratu et al. [24]	64/M	Acute upper abdominal pain, weight loss	US, endoscopy, CT	Adenocarcinoma	Body/3.2 × 1.9	Surgical resection of the lesion	—
23	2017	Al Warith et al. [25]	71/F	Left iliac fossa pain	CT, MRI, EUS	Mucinous neoplasia	Tail/2.4	Laparoscopic distal pancreatectomy, splenectomy	—
24	2018	Raymundo et al. [26]	36/M	Lumbar pain	CT, MRI, ERCP	Neuroendocrine tumor	Body tail/2.4 × 2.2	Distal pancreatectomy, splenectomy	After 6 months, the patient is in good condition, without abdominal symptoms.
25	2019	Lianyuan et al. [27]	63/M	Left upper abdominal pain and defecation unformed	CT, EUS	—	Head/10 × 5 × 5	Pancreaticoduodenectomy	Remained symptom free 2 years after surgery
26	2020	Zhou and Chen [28]	71/F	Slight pain on left upper abdomen	US, CT	Cystadenoma or adenocarcinoma	Head/3.2 × 3.0	Central pancreatectomy with pancreatojejunostomy	No complaints or recurrence of abdominal pain occurred in the next 3 years
27	2020	Jin et al. [29]	52/F	Epigastric pain	EUS, CT	Mucinous/serous cystic neoplasm	Body/4.6 × 4.6 × 3.4	Pancreaticojejunostomy	No recurrence after 10 months of follow‐up
28	2021	Langmaid et al. [30]	69/F	No symptoms	EUS, CT, MRI, PET	—	Head/1.1	Conservative management	Remained well and asymptomatic
29	2023	Li [31]	18/M	Physical examination	EUS, CT, MRI	—	Tail/6.4 × 5.1	Distal pancreatic tumor resection	No complications or recurrences were observed during the follow‐up period
30	2024	Lei et al. [32]	60/M	Intermittent dull pain, aggravated at night	CT, MRI	—	Head/5.8 × 7.5	Abdominal surgery	No tumor recurrence or metastasis was found in the postoperative follow‐up for 5 years
31	—	Present case	54/F	No symptoms	CT, MRI	Serous cystadenoma	Head/2.5 × 1.5 × 2.0	Laparoscopic pancreaticoduodenectomy	No complications on clinical follow‐up 4 months after surgery

Abbreviations: CT, computed tomography; ERCP, endoscopic retrograde cholangiopancreatography; EUS, endoscopic US; MRI, magnetic resonance imaging; US, ultrasonography.

Diagnostic imaging of pancreatic hemangioma is mostly confirmed by CT (27/34, 79.4%), followed by US or endoscopic US (EUS) (20/34, 58.8%), and MRI applied in recent cases (16/34, 47.17%). Other imaging tests such as angiography, endoscopy, endoscopic retrograde cholangiopancreatography (ERCP), abdominal plain film, cholangiography, and PET may also be found in a few cases. The site of the disease was most commonly located in the head of the pancreas (18/34, 52.9%), followed by the body‐tail (6/34, 17.6%), tail (5/34, 14.7%), body (4/34, 11.8%), and pancreatic neck (1/34, 2.9%). The maximum diameter of the lesion ranged from 0.5 to 20.0 cm. Under US examination, pancreatic hemangioma appeared as a hyperechoic mass with no or low velocity venous flow signal, which contrasts with the adequate blood supply of malignant tumors. On US, the hemangioma may show rapid enhancement but the clearance speed is slow, forming an obvious “fast in, slow out” sign [6, 7, 8, 11, 14, 15, 17, 18, 21, 23, 24, 25, 28, 29, 30, 31]. Since the patient in this case did not undergo US in our hospital, we were not able to observe this presentation. By CT and MRI, the tumor was usually presented as a classically rounded, well‐defined, and low‐density mass with a multilocular or honeycomb shape and no dilatation of the main pancreatic duct [6, 7, 9, 10, 11, 12, 13, 14, 15, 16, 17, 18, 19, 20, 21, 22, 23, 24, 25, 26, 27, 28, 29, 30, 31, 32]. In addition, some pancreatic hemangiomas reported in the literature presented as highly vascular masses, usually showing strong enhancement in the arterial phase of enhanced images. However, this finding was not confirmed in our case or in some other cases. The reason for this may be the lack of larger supplying vessels, slow blood flow within the tumor, and the absence of significant arteriovenous shunting, which may result in a low degree of enhancement. Also, there were different proportions of cystic and solid components in the tumor, which may lead to varying degrees of enhancement in the arterial phase [7, 8, 9, 27, 31]. Furthermore, in patients with pancreatic hemangioma, only a very small number of patients would show characteristic changes, and the vast majority of imaging diagnoses are difficult. Therefore, it is easy to confuse it with other pancreatic lesions (e.g., pseudocysts, serous cystadenomas, mucinous cystadenomas, and IPMN). However, through our case and review of the literature, the diagnosis of hemangioma cannot be excluded when imaging reveals a multilocular cystic tumor of the pancreas without invasion of adjacent structures, obstruction of the main pancreatic duct or other signs of malignancy such as lymph node metastasis, and enhancement is not significant. Pre‐surgical imaging is still essential to provide some indication and to rule out the malignancy of the lesion. If malignancy can be safely ruled out, the decision to operate must be based on a risk–benefit analysis. In some cases, close observation and regular follow‐up may be more beneficial.

Currently, the gold standard for the diagnosis of pancreatic hemangioma remains pathologic examination. Microscopically, hemangiomas consist predominantly of dilated abnormal sinus nodes lined with a single layer of vascular endothelial cells, which are not completely spaced by fibrous tissue forming spongy structures. Depending on the size of the vascular space, they can be capillary or spongy. On immunohistochemistry, CD31 and CD34 positivity indicates hemangiomas, lymphangiomas, and other benign vascular tumors, and the lymphatic endothelial marker D2‐40 negativity and lymphocyte deficiency help to rule out lymphangiomas [22, 27, 31]. Unlike the established observations for pediatric hemangioma, there are no definitive standards for the treatment of pancreatic hemangiomas in adults. However, surgical resection is usually recommended due to its risk of sudden bleeding and the uncertain differential diagnosis with epithelial tumors [23, 31]. The choice of surgical approach is based primarily on the location of the pancreatic hemangioma and is also influenced by the size of the tumor. So far, no cases of recurrence or metastasis have been found. Most patients achieved good clinical outcomes after surgical treatment.

In conclusion, pancreatic hemangioma in adults is a rare benign tumor without specific clinical symptoms and lacks typical imaging manifestations. Diagnosis is usually made postoperatively through histologic examination and immunohistochemical studies. However, careful evaluation of imaging characteristics through CT and MRI, combined with US and laboratory examination results, can provide valuable information for diagnosing pancreatic hemangioma and guiding management or treatment decisions.

## Author Contributions

**Jing Zhang:** conceptualization, data curation, formal analysis, resources, writing – original draft, writing – review and editing. **Jingjing Chen:** data curation, formal analysis, supervision. **Dongmei Zou:** data curation, formal analysis. **Xu Cao:** supervision, visualization, writing – review and editing.

## Funding

The authors have nothing to report.

## Ethics Statement

This study was performed in line with the principles of the Declaration of Helsinki.

## Consent

The patient gave her written informed consent for the publication of any identifying information/images in this case report.

## Conflicts of Interest

The authors declare no conflicts of interest.

## Data Availability

The datasets generated during the current study were available from the corresponding author on reasonable request.
